# Deep learning-based denoising in cardiac CT: effects on image quality, calcium scoring interchangeability, and reporting workflow

**DOI:** 10.3389/fradi.2026.1764357

**Published:** 2026-04-29

**Authors:** Daniel Wessling, Jan Magnus, Jan M. Brendel, Sebastian Werner, Patrick Krumm, Konstantin Nikolaou, Saif Afat, Andreas S. Brendlin

**Affiliations:** 1 Department of Diagnostic and Interventional Radiology, Friedrich-Alexander-University Erlangen, Erlangen, Germany; 2 Department of Diagnostic and Interventional Radiology, Eberhard-Karls University, Tuebingen, Germany; 3 Cardiovascular Imaging Research Center (CIRC), Department of Radiology, Massachusetts General Hospital, Harvard Medical School, Boston, MA, United States

**Keywords:** cardiac CT, coronary CT, deep learning, denoising, image quality, workflow

## Abstract

**Objectives:**

Ischemic heart disease is a major global health burden requiring timely diagnosis. Although coronary artery calcium (CAC) scoring and coronary computed tomography angiography (CCTA) are valuable tools, image noise can distort assessment. Deep learning-based denoising (DLD) algorithms may enhance quality, yet their impact on cardiac CT workflows remains unclear. This study evaluated DLD effects on CAC and CCTA image quality, clinical interchangeability, and workflow efficiency compared with iterative reconstruction (IR).

**Materials and methods:**

A retrospective analysis of 100 patients with CAC and CCTA scans from the same CT scanner was performed. IR and DLD reconstructions yielded 400 datasets, rated by two radiologists using a semiquantitative scoring system. Objective metrics (CT number stability, noise, contrast-to-noise ratio) were measured. Clinical interchangeability and Workflow efficiency were evaluated.

**Results:**

DLD showed significantly higher overall image quality than IR (*p* < 0.001), while preserving CT attenuation and improving noise and CNR. Although cardiac age classifications did not differ between reconstruction methods, Agatston scores were significantly higher in IR before manual correction (*p* < 0.001. After manual correction, Agatston scores were not significantly different between IR and DLD (*p* ≥ 0.158), supporting clinical comparability of the derived clinical metrics between the two approaches. DLD also reduced manual correction time (*p* < 0.001).

**Conclusions:**

The investigated DLD algorithm improves image quality and radiological workflows thus potentially enhancing overall patient care. Key limitations include the retrospective single-center design and inherent subjectivity in image-quality evaluation; therefore, findings should be confirmed in prospective studies with more objective measures.

## Introduction

Ischemic heart disease poses a significant global health burden, making the early identification of risk factors and symptoms paramount (1). Angina pectoris, a key indicator of potential coronary artery disease, often prompts individuals to seek medical care. Unfortunately, many patients delay consultation until symptoms become evident, often coinciding with the development of coronary stenosis—a condition that may necessitate invasive interventions (2). For symptomatic patients with a low to intermediate pre-test probability of requiring invasive intervention, excluding coronary stenosis is essential (3). Coronary artery calcium scoring (CAC) and coronary computed tomography angiography (CCTA) offer a valuable, non-invasive alternative to invasive coronary angiography for those with chronic, stable angina pectoris. CAC and CCTA excel in ruling out coronary calcifications and stenoses, utilizing tools like the Agatston score and cardiac age assessment for evaluation (4–6). While effective, image noise can negatively affect CAC and CCTA image quality, making clinical assessment more challenging (7). Fortunately, recent advances in deep learning algorithms show promise in noise reduction (8, 9).

Although deep learning-based denoising (DLD) has demonstrated robust reductions in image noise and improvements in quantitative image quality metrics, several methodological and perceptual limitations have to be considered. Alterations of image texture have been reported, occasionally resulting in a homogenized or artificial appearance when compared with conventional iterative reconstruction images. In addition, there is a theoretical concern that aggressive noise suppression may lead to partial smoothing of fine anatomical structures consequently impairing contrast details and potentially affecting diagnostic confidence in subtle findings. Prior investigations into deep learning-based reconstruction techniques have emphasized the importance of texture preservation and faithful representation of anatomical detail. Consequently, a critical appraisal of both the benefits and potential drawbacks of DLD is necessary to ensure that gains in image quality are not achieved at the expense of diagnostically relevant information.

However, the real-world implications of utilizing deep learning-based denoising on full-dose CCTA image quality and radiological workflows remain to be fully explored. Therefore, we aimed to assess how a deep learning-based denoising algorithm influences CAC and CCTA when compared to the established advanced modeled iterative reconstruction (IR) technique, specifically regarding subjective and objective image quality parameters and radiological workflow efficiency. We hypothesized that the DLD algorithm could help improve patient care by improving image quality and streamlining radiological workflows.

## Materials and methods

### Study design and patient population

This single-center, retrospective, single-institution study was approved by the local institutional review board with a waiver of informed consent (#167/2022BO2). The study was conducted in accordance with the ethical standards of the Declaration of Helsinki (1964, revised 2013). An *a priori* power analysis using the software solution G*Power (ver. 3.1.9.7, Franz Faul, University of Kiel, Germany) determined the required sample size (*f* = 0.18, *α* = 0.05, 1 − *β* = 0.95) to be 100 patients (10). We assessed all CTs from the same scanner for eligibility from 1 January 2021 to 1 January 2022. In the first step, all patients in this timeframe receiving scans different than CAC & CCTA were excluded (“no cardiac CT”). In the second step, only the most recent 100 CAC & CCTA patients were enrolled, and any additional CAC & CCTA patients were excluded (“overhead”). From the 100 patients enrolled, we collected sex, height, and weight from their clinical reports at the time of the procedure. We computed the body-mass index (BMI in kg/m^2^) from their height and weight. Furthermore, the patient's CTDI_vol_ (Computer Tomography dose index in mGy), DLP (dose-length product in mGy*cm), as well as the SSDE (size-specific dose estimate in mGy) were extracted using the dose-management software DoseM ® (Infinitt Europe GmbH, Frankfurt am Main, Germany).

### Image acquisition and reconstruction parameters

All images were acquired using the same third-generation multidetector dual-source CT scanner (SOMATOM Force; Siemens Healthineers, Erlangen, Germany). All patients were positioned head-first on their back with elevated arms. Electrocardiograms and echocardiograms were performed prior to imaging. Metoprolol was administered to patients without contraindications and heart rates above 60 bpm. For CAC scoring, a non-contrast scan was used (120 kV, 60 mAs, 3.2 pitch).

CCTA protocols were selected based on heart rate: ultra-fast flash for regular rates below 60 bpm, step-and-shoot with prospective ECG-gating (70%–80% diastole) for regular rates of 60–75 bpm, and helical acquisition with retrospective ECG-gating for higher/irregular rates. All patients without contraindications received sublingual nitroglycerin prior to CCTA scanning. The CCTA scans were contrast-enhanced using Imerone 400 (Bracco, Milan, Italy), and contrast medium arrival was estimated using a test bolus of 10 mL.

For the examination itself, 60 mL contrast medium, as well as a chaser of 50 mL saline were applied through a peripheral venous cannula by an automated power injector (CT Stellant, Medrad, Indianola, PA, USA) at a flow rate of 3 mL/s. Image acquisition utilized automated voltage and current modulation (CARE Dose 4D; 70–120 kV, reference 110 kV), with a 0.6 × 192 mm collimation and 0.25-second gantry rotation. Flash and helical modes used a pitch of 3.2, and step-and-shoot used Flex 0.25. All protocols had a 512 matrix size. Furthermore, all images were reconstructed using Advanced Modeled Iterative Reconstruction (IR) strength 2 (ADMIRE, Siemens Healthineers, Erlangen, Germany). A specialized medium-soft kernel (Sa36) with a slice thickness of 3 mm and an increment of 1.5 mm was used for CAC reconstructions. CCTA datasets were reconstructed using a vascular medium-soft kernel (Bv40) with a slice thickness of 0.75 mm and an increment of 0.5 mm. Additionally, all CAC and CCTA datasets were post-processed using a novel, commercially available, vendor-agnostic deep learning-based denoising (DLD) software solution (ClariAce ver 1.0.2, ClariPi Inc., Seoul, South Korea), with the contrast enhancement and noise blending settings fixed at 0. The model itself employs a Deep Convolutional Neural Network (CNN) based on a U-Net architecture. As detailed in validation studies, e.g., by Hong et al. (11) the model was trained using a combination of low-dose simulation data and high-quality target data. This hybrid training approach allows the network to learn effective noise subtraction while preserving structural edges and preventing overfitting.

### Subjective image quality comparison

A group member otherwise not associated with subjective image quality assessment created all possible dataset combinations per dataset (CAC, CCTA) and series (IR, DLD), including permutations (*n* = 2 combinations per dataset, *n* = 4 combinations per patient). Two radiologists with different experience levels in cardiac CT evaluation (rater 1 = 5 years, rater 2 = 8 years) independently rated all 400 dataset combinations for overall subjective image quality. All readings were performed in a blinded and randomized forced-choice setup using the freely available software solution ViewDex (ver. 3.0, Angelica, Sahlgrenska University Hospital, Gothenburg, Sweden) (12). Each combination was displayed simultaneously, with the reference series on the left and the rating series on the right. In each instance, points were awarded to the rating dataset for inferiority (−1 point), equality (0 points), or superiority (1 point) when compared to the reference dataset. Each series combination was initially displayed in a mediastinum window [Level 50 Hounsfield units (HU), Width 350 HU]. Individual adjustments were allowed for the readings. The overall image quality points were averaged per dataset and series to form a comparable semiquantitative mean subjective score, ranging from −1 (worst average performance in comparisons) to 1 (best average performance in comparisons).

### Objective image quality comparison

For objective image quality assessments, all series (IR, DLD) were loaded sequentially per dataset (CAC, CCTA) and patient into the open-source ImageJ distribution FIJI (ver. 1.53k, Wayne Rasband, National Institutes of Health-NIH, Maryland, USA) (13). The datasets were loaded in unison and linked at matching z-axis positions to ensure intraindividual conformity. In each dataset group (CAC/CCTA), the IR series was used to manually draw three non-overlapping regions of interest (ROI) with a diameter of 1 cm^2^ in homogenous areas of paraspinal muscle and cancellous tissue of vertebral bones. These reference tissues were chosen for their comparable CT attenuation to myocardium and calcifications. All ROIs were placed, excluding pathologies and artifacts. The program then conveyed those ROIs into the corresponding DLD series and performed place-consistent measurements of mean CT numbers in HU and their standard deviation (SD). The SD of HU was defined as noise. Furthermore, we computed a Contrast-to-Noise Ratio (CNR) between cancellous bone and paraspinal muscles.

### Clinical interchangeability and workflow analysis

We assessed the clinical interchangeability of the different series in coronary artery calcium scoring by comparing the Agatston score (14) and cardiac age (7) between the IR and the DLD series of the CAC datasets. CAC analysis was performed semi-automatically using a dedicated workstation (Siemens Syngo.Via version vb 60A_HS01, workflow coronary and CAC). All images were read in a blinded random order by a radiologist with eight years of CAC scoring experience. Using a threshold of 130 HU, calcifications were recognized automatically and manually corrected if necessary. Cardiac Age, Agatston score values and Risk Score groups were computed automatically for comparison before and after manual correction. The time expenditure for manual corrections in both series (IR, DLD) was measured to analyze potential workflow benefits.

### Statistical analysis

GraphPad Prism version 9.4 for Windows (GraphPad Software, San Diego, California, USA) was used for standard hypothesis testing and data illustration. Advanced Bayesian modeling was performed using Python (v3.x) with the PyMC and ArviZ libraries.

We assessed data distribution with the Shapiro–Wilk test, expressing normally distributed data as mean ± SD and non-normally distributed data as median and IQR. A mixed-effects model was utilized with Greenhouse-Geisser (for sphericity violations) and Holm-Šídák corrections (for type-1-error in multiple comparisons). Subjective image quality comparisons treated each dataset (CAC, CCTA) and series (IR, DLD) combination per patient as repeated measures. Similarly, we considered CT values, noise levels, and contrast-to-noise ratios across datasets and series as repeated measures. Effect sizes were calculated to quantify the magnitude of observed differences: Cohen's *d* was reported for parametric comparisons, while the effect size *r* $\left(r\;=\;\frac{\left|Z\right|}{\sqrt{N}}\right)$ was reported for non-parametric comparisons (Wilcoxon signed-rank tests).

To robustly assess clinical interchangeability and workflow reliability, we employed a Bayesian Gaussian-Copula Probabilistic Agreement Model. This approach models the dependency structure of paired measurements (e.g., IR vs. DLD) in a latent Gaussian space to estimate the probability of practical equivalence (*p_δ_*) within a defined tolerance (*δ* = 0.2). We report the posterior mean probability of agreement and the 95% Highest Density Interval (HDI).

Statistical significance for hypothesis testing was set at an adjusted *p*-value ≤0.05. Spearman rank correlation assessed intra-rater reliability and inter-rater agreement in subjective ratings, with correlation coefficients (*r*) classified as negligible (0–0.20), weak (0.21–0.40), moderate (0.41–0.60), strong (0.61–0.80), and very strong (0.81–1.00).

## Results

### Study population

**Figure 1:**
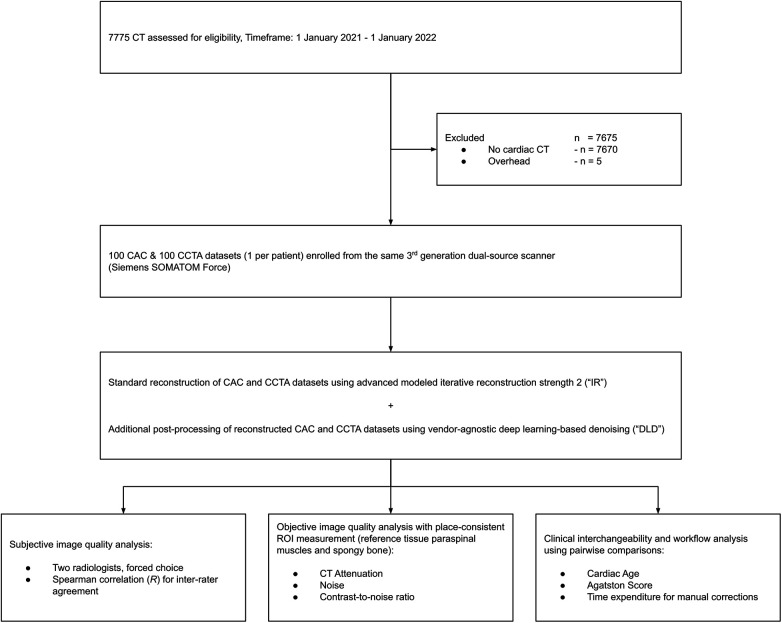
Study flowchart.

The initial database search revealed 7775 CTs between 1 January 2021 and 1 January 2022 from the same 3rd generation dual-source scanner. After exclusion according to our predefined criteria (*n* = 7,675), we enrolled 100 patients (mean age 60 ± 11 years, mean BMI 27 ± 3; 30 female). In our cohort, no patient received cardiac CT more than once. For further details about our study population, see Table 1. Figure 1 is a flowchart of the study process and patient enrollment.

**Table 1:** Study population and image acquisition parameters.

Group	Variable	Measure	Value	Protocol 1			Protocol 2			Protocol 3			Overall		
				Female	Male	Overall	Female	Male	Overall	Female	Male	Overall	Female	Male	Overall
Group	Variable n	Measure	Value	15	35	50	10	20	30	5	15	20	30	70	100
Population	age	Mean ± SD		60 ± 15	58 ± 11	59 ± 12	63 ± 8	58 ± 12	60 ± 11	64 ± 6	63 ± 12	63 ± 10	61 ± 12	59 ± 11	60 ± 11
	kg	Mean ± SD		74 ± 8	78 ± 8	77 ± 9	76 ± 9	81 ± 7	79 ± 8	75 ± 10	82 ± 5	81 ± 7	75 ± 8	80 ± 8	78 ± 8
	cm	Mean ± SD		169 ± 11	172 ± 11	171 ± 11	172 ± 12	176 ± 10	174 ± 11	166 ± 13	173 ± 10	171 ± 11	169 ± 12	173 ± 11	172 ± 11
	BMI	Mean ± SD		26 ± 3	27 ± 3	27 ± 3	26 ± 3	26 ± 2	26 ± 2	27 ± 1	28 ± 3	28 ± 2	26 ± 2	27 ± 3	27 ± 3
Prep 1	Beta blocker	n	0 mg	8	18	26	3	8	11	1	7	8	12	33	45
		n	2.5 mg				1	1	2		1	1	1	2	3
		n	5 mg	3	11	14	4	8	12	1	5	6	8	24	32
		n	7.5 mg					1	1	1		1	1	1	2
		n	10 mg	4	6	10	2	2	4	2	2	4	8	10	18
	HR	Mean ± SD		67 ± 4	67 ± 5	67 ± 5	85 ± 9	85 ± 8	85 ± 8	78 ± 9	114 ± 15	114 ± 15	85 ± 8	114 ± 13	114 ± 12
Image	kV	n	120	15	35	50	10	20	30	5	15	20	30	70	100
	mAs	Mean ± SD		72 ± 48	83 ± 24	80 ± 33	69 ± 29	86 ± 27	80 ± 28	88 ± 29	111 ± 80	105 ± 70	74 ± 39	90 ± 44	85 ± 43
	CTDIvol	Mean ± SD		0.96 ± 0.18	1.00 ± 0.19	0.99 ± 0.19	1.02 ± 0.18	1.04 ± 0.16	1.03 ± 0.16	1.34 ± 0.09	1.39 ± 0.14	1.38 ± 0.13	1.04 ± 0.21	1.09 ± 0.23	1.08 ± 0.23
	DLP	Mean ± SD		18.78 ± 3.85	19.61 ± 4.28	19.36 ± 4.13	19.79 ± 3.46	20.21 ± 3.08	20.07 ± 3.16	25.70 ± 1.66	26.46 ± 2.62	26.27 ± 2.40	20.27 ± 4.19	21.25 ± 4.54	20.96 ± 4.44
	SSDE	Mean ± SD		1.33 ± 0.15	1.36 ± 0.16	1.35 ± 0.15	1.42 ± 0.13	1.43 ± 0.12	1.42 ± 0.12	1.81 ± 0.03	1.82 ± 0.05	1.82 ± 0.05	1.44 ± 0.22	1.48 ± 0.22	1.46 ± 0.22
Prep 2	Nitroglycerine	n	0 sprays		5	5	3	1	4		6	6	3	12	15
		n	1 spray		2	2	1	1	2				1	3	4
		n	2 sprays	14	27	41	6	17	23	5	9	14	25	53	78
		n	3 sprays	1	1	2							1	1	2
		n	4 sprays					1	1					1	1
Image acquisition parameters: CCTA	kV (n)	n	70	9	6	15	5	5	10		3	3	14	14	28
		n	80	3	12	15	4	10	14	1	4	5	8	26	34
		n	90	2	10	12					4	4	2	14	16
		n	100	1	4	5		1	1	1		1	2	5	7
		n	110							1		1	1		1
		n	120		3	3	1	4	5	2	4	6	3	11	14

**mAs**	**Mean ± SD**	525 ± 100	537 ± 95	533 ± 95	496 ± 92	483 ± 92	487 ± 91	439 ± 81	507 ± 115	490 ± 109	501 ± 96	515 ± 100	511 ± 99
**CTDIvol**	**Mean ± SD**	2.01 ± 0.57	2.14 ± 0.66	2.10 ± 0.63	11.48 ± 3.99	11.91 ± 3.46	11.77 ± 3.58	50.79 ± 5.53	53.54 ± 8.48	52.85 ± 7.80	13.29 ± 17.85	15.95 ± 20.66	15.15 ± 19.81
**DLP**	**Mean ± SD**	30.38 ± 9.45	32.65 ± 10.99	31.97 ± 10.51	167.91 ± 56.72	174.11 ± 49.17	172.04 ± 50.90	813.53 ± 70.11	847.03 ± 108.90	838.65 ± 99.98	206.75 ± 286.02	247.58 ± 325.95	235.33 ± 313.64
**SSDE**	**Mean ± SD**	39.72 ± 17.95	44.67 ± 22.40	43.19 ± 21.10	239.63 ± 122.34	250.89 ± 103.86	247.14 ± 108.35	1,312.37 ± 226.26	1,437.33 ± 336.51	1,406.09 ± 311.93	318.47 ± 473.82	402.02 ± 574.98	376.95 ± 545.59

BMI, Body Mass Index (kg/cm²); HR, Heart Rate in beats per minute; CTDIvol, Computed Tomography Dose Index (mGy); DLP, Dose Length Product (mGy*cm); ED, Effective Radiation Dose (mSv); Protocol 1, Ultra-fast Flash Scan; Protocol 2, Prospective ECG-gated Scan; Protocol 3, Helical Scan with Restrospective ECG-gating.

### Subjective image quality comparison

**Figure 2:**
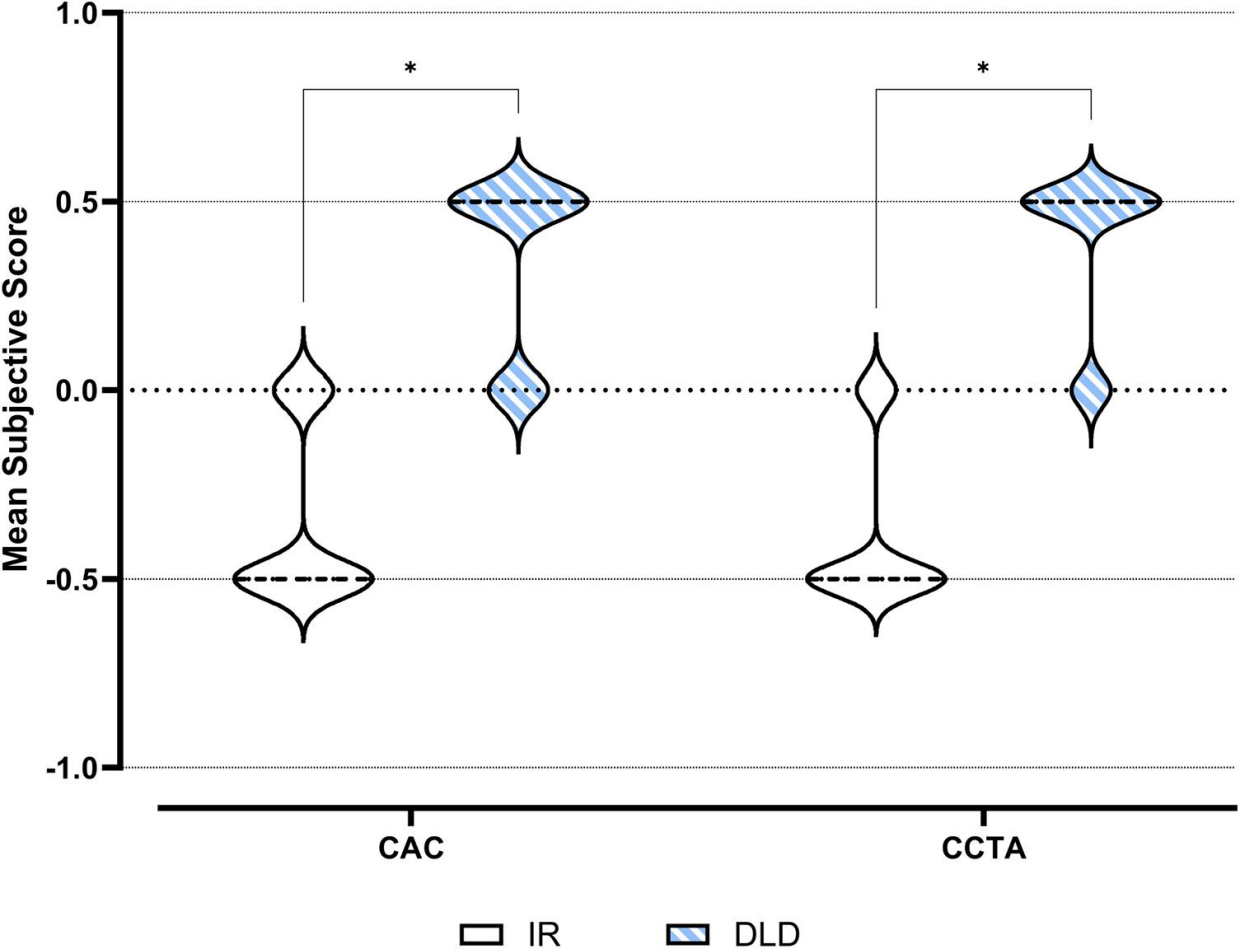
Semiquantitative assessment of subjective image quality: violin plots with pairwise comparisons. ns, not significant; *, significant.

We measured very strong intra-rater reliability (*r* = 1.00) and strong inter-rater agreement (*r* ≥ 0.79). See Table 2 for rater-specific results and the complete correlation matrix. Mixed effects analysis of the pooled subjective quality scores showed significant interactions between the semiquantitative score points [*F*(1.00; 99.0) = 4.66; *p* = 0.033; *ε* = 1.000]. In pairwise *post hoc* comparisons, the DLD series received significantly higher subjective scores than the IR series in both datasets (each *p* < 0.001). Figure 2 visualizes data distribution and pairwise comparisons of the pooled subjective score points, and Table 3 features further numerical results and the pooled pairwise comparison matrix.

**Table 2:** Subjective image quality ratings and intra-rater reliability and inter-rater agreement (spearman's Rho).

Dataset	Series	Rater	mean ± SD	Spearman Correlation Coefficient (*r)* with 95% CI			
				Original		Denoising	
				Rater 1	Rater 2	Rater 1	Rater 2
CAC	IR	Rater 1	−0.39 ± 0.49	1.00	0.79 (0.72 to 0.84)	0.64 (0.55 to 0.72)	0.50 (0.39 to 0.60)
		Rater 2	−0.31 ± 0.54	0.79 (0.72 to 0.84)	1.00	0.50 (0.39 to 0.60)	0.39 (0.27 to 0.51)
	DLD	Rater 1	0.39 ± 0.49	0.64 (0.55 to 0.72)	0.50 (0.39 to 0.60)	1.00	0.79 (0.72 to 0.84)
		Rater 2	0.31 ± 0.54	0.50 (0.39 to 0.60)	0.39 (0.27 to 0.51)	0.79 (0.72 to 0.84)	1.00
CCTA	IR	Rater 1	−0.41 ± 0.49	1.00	0.90 (0.86 to 0.92)	0.69 (0.61 to 0.76)	0.62 (0.53 to 0.70)
		Rater 2	−0.37 ± 0.52	0.90 (0.86 to 0.92)	1.00	0.62 (0.53 to 0.70)	0.56 (0.45 to 0.65)
	DLD	Rater 1	0.41 ± 0.49	0.69 (0.61 to 0.76)	0.62 (0.53 to 0.70)	1.00	0.90 (0.86 to 0.92)
		Rater 2	0.37 ± 0.52	0.62 (0.53 to 0.70)	0.56 (0.45 to 0.65)	0.90 (0.86 to 0.92)	1.00

CAC, coronary artery calcium scoring; CCTA, coronary computed tomography angiography; IR, iterative reconstruction; DLD, deep learning-based denoising; SD, standard deviation; 95% CI, 95% confidence interval.

**Table 3:** Pooled subjective image quality metrics, adjusted two-tailed pairwise comparison (*p*) and effect sizes (*d*).

Dataset	Series		*p*-Value				*d*-Value			
		mean ± SD	CAC		CCTA		CAC		CCTA	
		Pooled	IR	DLD	IR	DLD	IR	DLD	IR	DLD
CAC	IR	−0.35 ± 0.23	n/a	<0.001	0.406	<0.001	n/a	3,043	0,182	3,360
	DLD	0.35 ± 0.23	<0.001	n/a	<0.001	0.406	3,043	n/a	3,360	0,182
CCTA	IR	−0.39 ± 0.21	0,406	<0.001	n/a	<0.001	0,182	3,360	n/a	3,714
	DLD	0.39 ± 0.21	<0.001	0.406	<0.001	n/a	3,360	0,182	3,714	n/a

CAC, Coronary Artery Calcium Scoring; CCTA, Coronary Computed Tomography Angiography; IR, Iterative Reconstruction; DLD, Deep Learning-based Denoising; SD, Standard Deviation; n/a, not applicable.

### Objective image quality comparison

**Figure 3:**
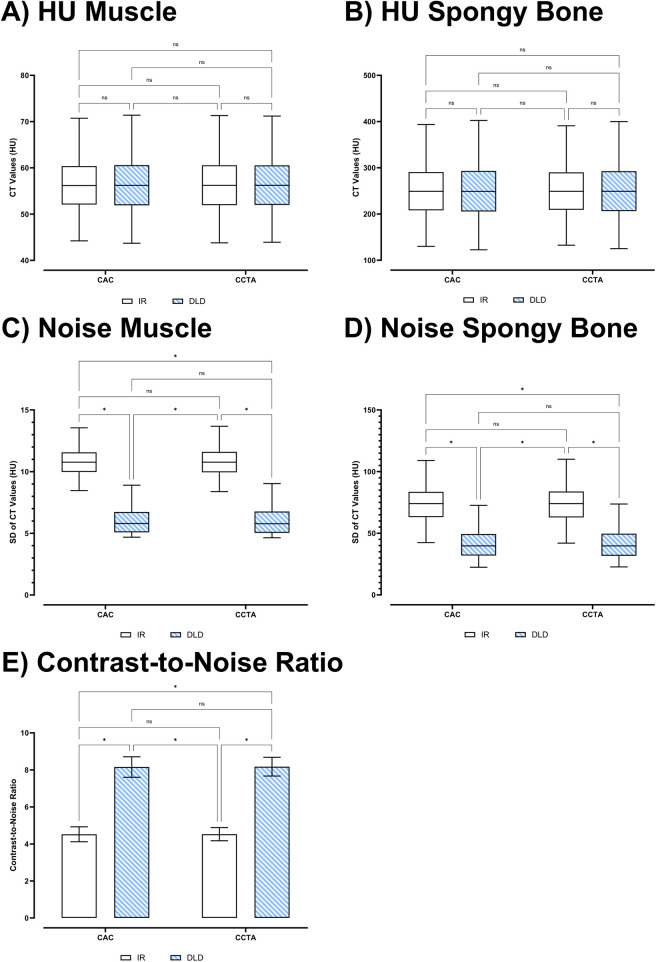
Data distribution and pairwise comparisons of objective image quality metrics: CT numbers of **(A)** muscle and **(B)** cancellous bone, noise of **(C)** muscle and **(D)** cancellous bone, and **(E)** contrast-to-noise ratios. ns, not significant; *, significant.

Mixed effects analysis showed significant interactions for the objective image quality metrics (HU, SD, CNR) of the different series and datasets [*F*(1.01; 99.5) = 4.662; *p* < 0.001; *ε* = 0.122]. In the corrected pairwise *post-hoc* tests, there were no significant differences between the CT values for paraspinal muscles (*p* ≥ 0.311) and the CT values of cancellous bone tissue (*p* = 0.999) in each CT dataset. For both datasets, noise was significantly lower in the DLD series than in the IR series (each *p* < 0.001). CNR metrics mirrored these results with significantly higher CNR for both DLD series than their IR counterparts (each *p* < 0.001). Figure 3 visualizes the data distributions and pairwise comparisons of the objective image quality metrics, and Table 4 has further numerical objective image quality metrics and pairwise comparisons.

**Table 4:** Objective image quality metrics, corrected two-tailed pairwise comparisons (p), and effect sizes (d).

Attribute	Dataset	Series	mean ± SD	*p*-Value				*d*-Value			
				CAC		CCTA		CAC		CCTA	
				IR	DLD	IR	DLD	IR	DLD	IR	DLD
HU Muscle	CAC	IR	56.42 ± 5.89	n/a	>0.999	0.962	0.311	n/a	0,005	0,007	0,008
		DLD	56.45 ± 6.15	>0.999	n/a	0.494	0.869	0,005	n/a	0,002	0,003
	CCTA	IR	56.46 ± 6.12	0.962	0.494	n/a	0.981	0,007	0,002	n/a	0,002
		DLD	56.47 ± 6.06	0.311	0.869	0.981	n/a	0,008	0,003	0,002	n/a
HU Cancellous Bone	CAC	IR	251.34 ± 58.62	n/a	>0.999	>0.999	>0.999	n/a	0,001	0,002	0,003
		DLD	251.38 ± 62.19	>0.999	n/a	>0.999	>0.999	0,001	n/a	0,001	0,002
	CCTA	IR	251.43 ± 57.43	>0.999	>0.999	n/a	>0.999	0,002	0,001	n/a	0,002
		DLD	251.52 ± 61.07	>0.999	>0.999	>0.999	n/a	0,003	0,002	0,002	n/a
Noise Muscle	CAC	IR	10.81 ± 1.13	n/a	<0.001	>0.999	<0.001	n/a	4,440	0,009	4,362
		DLD	6.01 ± 1.03	<0.001	n/a	<0.001	>0.999	4,440	n/a	4,343	0,000
	CCTA	IR	10.82 ± 1.18	>0.999	<0.001	n/a	<0.001	0,009	4,343	n/a	4,270
		DLD	6.01 ± 1.07	<0.001	>0.999	<0.001	n/a	4,362	0,000	4,270	n/a
Noise Cancellous Bone	CAC	IR	74.04 ± 14.88	n/a	<0.001	0.961	<0.001	n/a	2,469	0,005	2,447
		DLD	41.37 ± 11.35	<0.001	n/a	<0.001	0.999	2,469	n/a	2,450	0,003
	CCTA	IR	74.11 ± 15.11	0.961	<0.001	n/a	<0.001	0,005	2,450	n/a	2,428
		DLD	41.41 ± 11.59	<0.001	0.999	<0.001	n/a	2,447	0,003	2,428	n/a
CNR	CAC	IR	4.52 ± 0.40	n/a	<0.001	>0.999	<0.001	n/a	7,569	0,027	7,986
		DLD	8.16 ± 0.55	<0.001	n/a	<0.001	0.875	7,569	n/a	7,875	0,038
	CCTA	IR	4.53 ± 0.35	>0.999	<0.001	n/a	<0.001	0,027	7,875	n/a	8,345
		DLD	8.18 ± 0.51	<0.001	0.875	<0.001	n/a	7,986	0,038	8,345	n/a

HU, denoting CT numbers in HU; CNR, Contrast-to-Noise Ratio; CAC, Coronary Artery Calcium Scoring; CCTA, Coronary Computed Tomography Angiography; IR, Iterative Reconstruction; DLD, Deep Learning-based Denoising; SD, standard deviation; n/a, not applicable.

### Clinical interchangeability and workflow analysis

**Figure 4:**
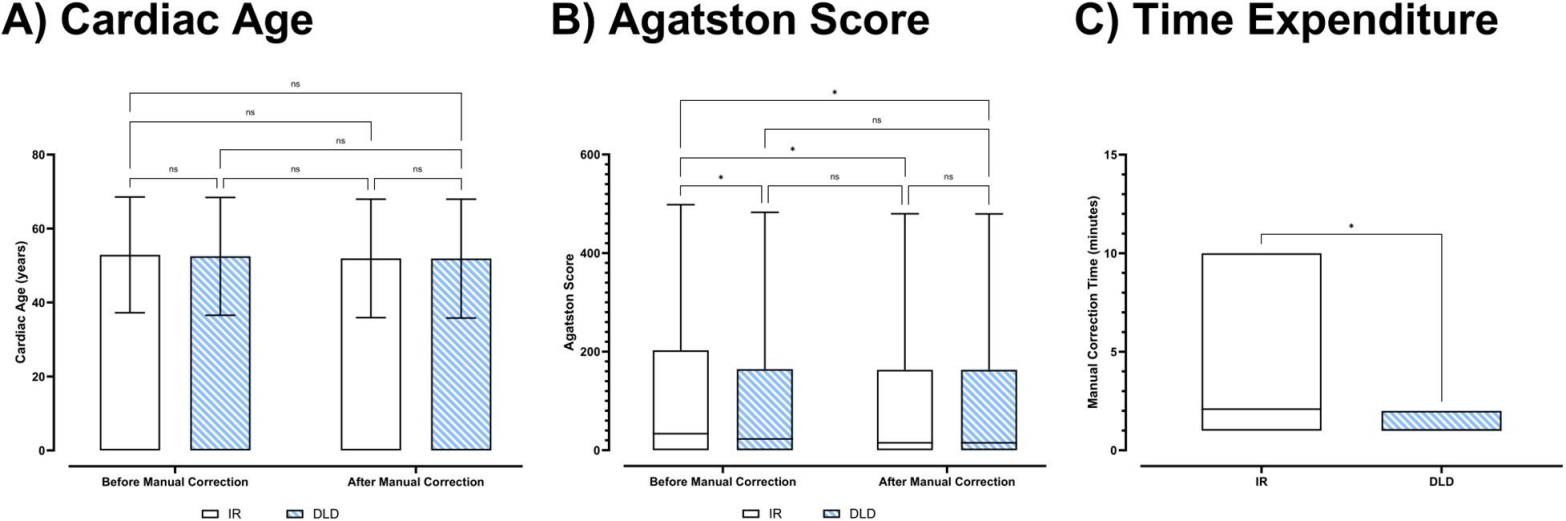
Data distribution and pairwise comparisons of clinical interchangeability and workflow metrics: **(A)** cardiac Age, **(B)** agatston score, and **(C)** time expenditure for manual corrections. ns, not significant; *, significant.

**Figure 5:**
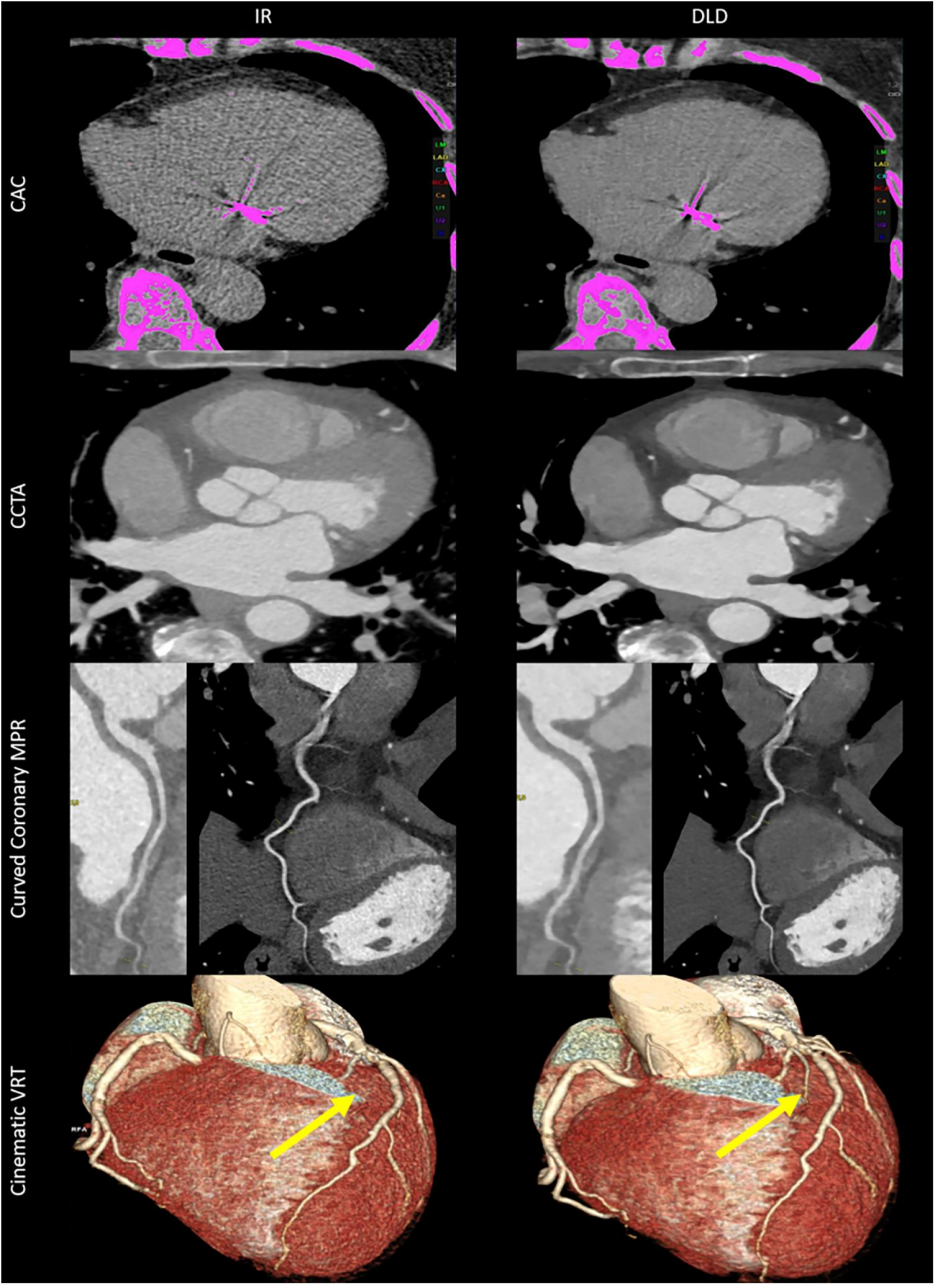
Illustrative overview images of the CAC and CCTA datasets using IR and DLD. The top row shows CAC images with significantly more noise IR, being misclassified as calcifications before manual corrections (pink overlay). DLD has fewer calcifications, translating to less manual corrections. The second row shows significant image quality improvements by DLD in CCTA, and the third row illustrates how image quality improvement translates into improved curved coronary multiplanar reconstructions (MPR). The bottom row shows cinematic volume renderings of the same patient, where the diagonal branch (yellow arrow) would not be identified and rendered correctly in the IR datasets due to high image noise.

Mixed effects analysis showed significant interactions for the cardiac age and Agatston score results of the different series (IR, DLD) before and after manual correction [*F*(1.35; 134.00) = 10.6; *p* < 0.001; *ε* = 0.450]. In the corrected pairwise *post hoc* tests, there were no significant differences in the cardiac age assessments of the series (IR, DLD) before and after manual correction (*p* ≥ 0.181). However, the Agatston score results of the IR series before manual correction were significantly higher than after manual correction (*p* = 0.005). In the DLD series, there were no significant differences between the Agatston score results before and after manual correction (*p* = 0.723). Furthermore, there were no differences between the Agatston score results of DLD before manual correction, DLD after manual correction, and IR after manual correction (*p* ≥ 0.158). Consecutively, the time expenditure for manual corrections was significantly shorter in the DLD series than in the IR series (*p* < 0.001). Figure 4 shows the data distributions of the clinical interchangeability and workflow analysis metrics, and Table 5 has further numerical clinical interchangeability and workflow analysis metrics with pairwise comparisons. Figure 5 illustrates superior image quality by DLD (right) compared to IR (left) in both CAC and CCTA datasets. DLD reduces noise, leading to fewer false-positive calcifications (first row), improved CCTA visualization (second row), and clearer curved coronary artery multiplanar reformations (“MPR”; third row). DLD also enables accurate rendering of smaller vessels (yellow arrows) obscured by IR noise (fourth row).

**Table 5:** Clinical interchangeability with workflow metrics, corrected two-tailed pairwise comparisons (p), effect sizes (d/r), and Bayesian Gaussian-copula agreement (BCGA).

Attribute	Dataset	Series		*p*-Value				d/r-Value				BGCA with 95% Highest Density Intervals			
			mean ± SD/median + IQR	BMC		AMC		BMC		AMC		BMC		AMC	
				IR	DLD	IR	DLD	IR	DLD	IR	DLD	IR	DLD	IR	DLD
Cardiac age	BMC	IR	53 ± 16	n/a	0,998	0,810	0,754	n/a	0,063	0,063	0,063	1.000 (1.000 to 1.000)	0.366 (0.210 to 0.531)	0.552 (0.406 to 0.699)	0.367 (0.200 to 0.524)
		DLD	52 ± 16	0,998	n/a	0,847	0,760	0,063	n/a	0,000	0,000	0.364 (0.204 to 0.520)	1.000 (1.000 to 1.000)	0.983 (0.973 to 0.992)	1.000 (1.000 to 1.000)
	AMC	IR	52 ± 16	0,810	0,847	n/a	0,496	0,063	0,000	n/a	0,000	0.554 (0.408 to 0.700)	0.983 (0.973 to 0.992)	1.000 (1.000 to 1.000)	0.983 (0.974 to 0.992)
		DLD	52 ± 16	0,754	0,760	0,496	n/a	0,063	0,000	0,000	n/a	0.364 (0.198 to 0.515)	1.000 (1.000 to 1.000)	0.983 (0.973 to 0.991)	1.000 (1.000 to 1.000)
Agatston score	BMC	IR	114 ± 140	n/a	0,018	0,026	0,022	n/a	0,128	0,134	0,134	1.000 (1.000 to 1.000)	0.795 (0.718 to 0.873)	0.902 (0.857 to 0.945)	0.497 (0.331 to 0.648)
		DLD	97 ± 126	0,018	n/a	>0.999	>0.999	0,128	n/a	0,008	0,008	0.795 (0.715 to 0.870)	1.000 (1.000 to 1.000)	0.996 (0.994 to 0.999)	0.935 (0.901 to 0.967)
	AMC	IR	96 ± 128	0,026	>0.999	n/a	0,425	0,134	0,008	n/a	0,000	0.902 (0.857 to 0.945)	0.996 (0.994 to 0.999)	1.000 (1.000 to 1.000)	0.877 (0.819 to 0.927)
		DLD	96 ± 128	0,022	>0.999	0,425	n/a	0,134	0,008	0,000	n/a	0.495 (0.332 to 0.652)	0.935 (0.900 to 0.966)	0.876 (0.821 to 0.929)	1.000 (1.000 to 1.000)
Risk classification	BMC	IR	1 (0–2)	n/a	0,141	0,276	0,276	n/a	0,264	0,141	0,264	1.000 (1.000 to 1.000)	0.793 (0.708 to 0.868)	0.991 (0.987 to 0.995)	0.793 (0.708 to 0.868)
		DLD	1 (0–2)	0,141	n/a	>0.999	>0.999	0,264	n/a	0,142	0,000	0.793 (0.712 to 0.872)	1.000 (1.000 to 1.000)	0.973 (0.958 to 0.986)	1.000 (1.000 to 1.000)
	AMC	IR	1 (0–2)	0,276	>0.999	n/a	>0.999	0,141	0,142	n/a	0,141	0.991 (0.987 to 0.995)	0.973 (0.958 to 0.986)	1.000 (1.000 to 1.000)	0.973 (0.958 to 0.986)
		DLD	1 (0–2)	0,276	>0.999	>0.999	n/a	0,264	0,000	0,141	n/a	0.793 (0.712 to 0.872)	1.000 (1.000 to 1.000)	0.973 (0.958 to 0.986)	1.000 (1.000 to 1.000)
TE	AMC	IR	2.1 ± 1.80	n/a	n/a	n/a	< 0.001	n/a	n/a	n/a	0,862	n/a	n/a	n/a	n/a
		DLD	1.0 ± 0.14	n/a	n/a	< 0.001	n/a	n/a	n/a	0,862	n/a	n/a	n/a	n/a	n/a

TE, time expenditure for manual corrections; BMC/AMC, before/after manual corrections; IR, iterative reconstruction; DLD, deep learning-based denoising; SD, standard deviation; n/a, not applicable.

## Discussion

Ischemic heart disease is a significant health issue, highlighting the importance of early risk factors and symptom identification. CAC and CCTA offer non-invasive alternatives to traditional coronary angiography for assessing patients with stable angina. Individual risks are effectively measured through cardiac age and the Agatston score, though image noise can affect quality and clinical interpretation. Recent DLD algorithms improve noise reduction without losing detail, unlike traditional IR2 reconstructions, yet their impact on cardiac CT image quality and workflows requires further workup. Our research aims to assess DLD's effect on image quality and clinical utility vs. standard IR images, including workflow efficiency and time needed for manual CAC scoring adjustments. We hypothesize that DLD could enhance patient care through improved image quality and more streamlined radiological workflows.

Our results showed that DLD significantly improved subjective image quality in CAC scoring and CCTA. Radiologists consistently preferred DLD images over standard IR images, suggesting DLD could improve visual clarity and diagnostic confidence. These findings support existing literature demonstrating the benefits of deep learning-based denoising for cardiac CT image quality, as described by Benz et al. (7). Similarly, De Santis et al. (9) and Jeon et al. (15) also found improved subjective image quality with deep learning-based reconstruction in CCTA. However, they used vendor-specific solutions, while our study demonstrates similar benefits with a more agile vendor-agnostic post-processing approach. Only Hong et al. used a similar approach, but our work uniquely evaluates both CCTA and CAC datasets (16). In synopsis, our findings further support the potential of deep learning to enhance subjective image quality across various cardiac CT applications.

In objective quality comparisons, DLD preserved mean CT attenuation values in CAC and CCTA, ensuring accurate tissue representation. The methodologies of multiple image quality studies corroborate the necessity to prove CT attenuation comparability as a baseline result when investigating novel image reconstruction algorithms (17–19). DLD significantly reduced image noise and improved contrast-to-noise ratios. The improvement is especially important in smaller coronary branches where accurate assessment of soft plaques impacts treatment decisions, as these lesions are linked to worse outcomes following revascularization (20). Our findings are consistent with existing research. Similar to Ahn et al. (21) and Hong et al. (16), we observed significant reductions in noise and improved objective image quality metrics with DLD in cardiac CT. Nishii et al.'s study further supports these positive results (22). In summary, there is substantial evidence for objective image quality metric improvements when using deep learning-based techniques in cardiac CT.

While DLD improves image quality, ensuring diagnostic equivalence to standard IR is crucial. Cardiac age and Agatston score values are key clinical parameters allowing comprehensive risk assessment (23). We found no significant classification differences between the series (IR, DLD) before and after manual correction when conducting pairwise comparisons of cardiac age, likely due to the nature of the classification system using age groups that are not sensitive to more minor Agatston score value changes. However, we observed significant differences in Agatston scores between IR and DLD images before manual correction. IR scores were initially overestimated due to threshold-based misclassification of noise speckles as coronary calcifications. After manual correction, this difference disappeared, demonstrating clinical interchangeability. Concordantly, the complication of quantitative cardiac CT by image noise has been previously reported by McCollough et al. (24) and corroborated by Yu et al. (25). However, these publications focused on standardizing scan protocols and CAC scoring on non-cardiac chest CT instead of clinical interchangeability when using novel DLD rather than IR. In synopsis, our results demonstrate a clear benefit for DLD because of significantly reduced noise misclassifications before manual correction and clinical endpoint interchangeability after manual correction.

Finally, a consecutive key benefit of the DLD's noise reduction was the significant decrease in time expenditure for manual corrections in Agatston score assessments. While direct workflow studies are rare, our results align with existing research demonstrating that DLD improves image quality and diagnostic confidence. Studies by Cao et al. (26), Kobayashi et al. (27), and Schottwell et al. (28) support this finding, showing improved spatial resolution, contrast-to-noise ratios, and reader confidence with DLD in various CT applications. As the demand for cardiac CT grows, DLD's potential to improve image quality, maintain clinical accuracy, and streamline workflow offers a significant advantage for cardiovascular radiologists.

This study has several limitations. First, its retrospective, single-center design introduces potential selection bias. While our sample size was sufficient for the study's aims, future multicenter studies with more extensive, diverse populations with potential subgroup analyses are needed to improve statistical power further and validate our findings. Second, subjectivity is inherent in image quality ratings. We used a forced-choice setup and semiquantitative scoring to mitigate this limitation. Although we found good reliability and agreement, the reader count of only two and the potential rater bias might limit the generalizability of image quality assessment. Third, our objective image quality analysis used limited ROIs, and future studies could expand these for a broader exploration of tissues and three-dimensional noise properties. Fourth, we used the cardiac age and the Agatston score as the primary metrics of diagnostic interchangeability. While well-established and clinically relevant, they may only capture some clinically significant aspects. Further work could include evaluating stenosis grading, plaque characteristics, and vessel visualization. Modern deep-learning segmentation tools would allow comprehensive volumetric noise assessment and automated volumetric tissue segmentation to capture heterogeneity more fully. While the DLD software itself is vendor-agnostic and therefore capable of processing DICOM input from any source, our study only validated its performance on a Siemens SOMATOM Force scanner. Thus, identical performance characteristics on hardware from other vendors cannot be guaranteed without specific validation. Finally, our specific hardware and software setup may limit the generalizability of our results, and future research should investigate DLD's impact across diverse technology setups. In our study reconstructions were not tested against algorithms with higher IR strengths or deep learning based IR. Therefore, a comparison against different reconstruction algorithms will be necessary to verify the potential of the DLD algorithm. We conclude that the investigated DLD algorithm improves calcium scoring and image quality through streamlined radiological workflows. In cardiac CT, image noise reduction and improved visualization of coronary plaques could potentially increase diagnostic confidence and reduce the need for extensive manual corrections. Future prospective studies are needed to confirm whether these advantages translate into measurable patient-centered outcome benefits.

## Data Availability

Data are available from the corresponding author upon reasonable request. Requests to access these datasets should be directed to andreas.brendlin@med.uni-tuebingen.de.
